# Postoperative controls of ventilation tubes in children by general practitioner or otolaryngologist? Study protocol for a multicenter randomized non-inferiority study (The ConVenTu study)

**DOI:** 10.1186/s13063-020-04849-3

**Published:** 2020-11-23

**Authors:** Bjarne Austad, Ann Helen Nilsen, Anne-Sofie Helvik, Grethe Albrektsen, Ståle Nordgård, Wenche Moe Thorstensen

**Affiliations:** 1 Department of Public Health and Nursing, Norwegian University of Science and Technology (NTNU), Trondheim, Norway; 2 Øya Medical Centre, Trondheim, Norway; 3 Department of Otolaryngology, Head and Neck Surgery, St. Olavs Hospital, Trondheim, Norway; 4 Department of Neuromedicine and Movement Science, NTNU, Trondheim, Norway

**Keywords:** Otitis media, Ventilation tubes, Primary care, Postoperative care

## Abstract

**Background:**

Otitis media with effusion is the major cause of acquired hearing problems in children. Some of the affected children need surgery with ventilation tubes in the tympanic membrane to reduce ear complaints and to improve hearing, middle ear function, and health-related quality of life. This is one of the most common ambulatory surgeries performed on children. Postoperative controls are needed to assess that the tubes are functional, to evaluate whether hearing loss has been improved, and to handle potential complications. The follow-up may continue for years and are usually done by otolaryngologists. Nevertheless, there exist no evidence-based guidelines concerning the level of expertise needed for postoperative controls of the ventilation tubes. The aim of this protocol is to describe the ConVenTu study that evaluates whether postoperative controls performed by general practitioners (GPs) represent a safe and sufficient alternative to controls performed by otolaryngologists.

**Methods/design:**

Multicenter randomized non-inferiority study conducted in clinical settings in seven hospitals located in Norway. Discharged children with ventilation tubes, aged 3–10 years, are allocated randomly to receive postoperative controls by either an otolaryngologist at the hospital where they had ventilation tube surgery or their regular GP. Study participants are enrolled consecutively until 200 patients are included in each group. Two years after surgery, we will compare the pure tone average of hearing thresholds (primary endpoint) and middle ear function, complication rate, health-related quality of life and the parents’ evaluations of the postoperative care (secondary endpoints).

**Discussion:**

This protocol describes the first randomized non-inferiority study of GPs performing postoperative controls after surgery with ventilation tubes. Results from this study may be utilized for deriving evidence-based clinical practice guidelines of the level of postoperative controls after ventilation tube surgery which is safe and sufficient.

**Trial registration:**

ClinicalTrials.govNCT02831985. Registered on 13 July 2016

## Administrative information

**Table:** 

Title {1}	Postoperative controls of ventilation tubes in children by general practitioner or otolaryngologist? Study protocol for a multicenter randomized non-inferiority study (the ConVenTu study)
Trial registration {2a and 2b}	Postoperative controls of ventilation tubes in children by general practitioner or otolaryngologist? (The ConVenTu study). Identifier: NCT02831985. Registered 13 July 2016, https://clinicaltrials.gov/ct2/show/NCT02831985?term=ventilation+tube&cntry=NO&city=Trondheim&draw=2&rank=1
Protocol version {3}	Oct 23rd 2020
Funding {4}	1. The Joint Research Committee between St. Olavs Hospital and the Faculty of Medicine, Norwegian University of Science and Technology (NTNU) (Felles forskningsutvalg) 2. The Liaison Committee for Education, Research and Innovation, The Central Norway Regional Health Authority (Samarbeidsorganet)
Author details {5a}	Bjarne Austad, MD PhD (1, 2) Ann Helen Nilsen, R.N. PhD (3, 4) Prof Anne-Sofie Helvik, R.N. Dr. Philos (1) Prof Grethe Albrektsen, Cand. Scient, Dr. Philos (1) Prof Ståle Nordgård, MD Dr. Med (3, 4) Wenche Moe Thorstensen, MD Ph. D (3, 4) (1) Dept. of Public Health and Nursing, Norwegian University of Science and Technology (NTNU), Norway (2) Øya Medical Centre, Trondheim, Norway (3) Dept. of Otolaryngology, Head and Neck Surgery St. Olavs Hospital, Trondheim, Norway (4) Dept. of Neuromedicine and Movement Science, NTNU, Norway
Name and contact information for the trial sponsor {5b}	Prof Jorunn Helbostad, Head of Dept. of Neuromedicine and Movement Science, NTNU, Norway
Role of sponsor {5c}	The sponsor is responsible for the study. Neither the sponsor nor the funding body have taken any active part in the study. They have no ultimate authority over the design of the study, analysis, interpretation of data, writing the manuscript nor the decision to submit the report for publication.

## Background

Otitis media with effusion, defined as middle-ear effusion without acute signs of infection, is the major cause of acquired hearing problems in children [1]. Some of the affected children need surgery to reduce ear complaints and to improve hearing, middle ear function, and health-related quality of life (HRQoL) [2, 3]. During surgery, a small incision is made in the tympanic membrane to drain the fluid from the middle ear. Then a tiny ventilation tube (VT), also called tympanostomy tube or grommet, is placed in the opening to keep the middle ear ventilated and prevent fluid from accumulating again. Indications for surgery are persisting otitis media with effusion or recurrent otitis media [3, 4]. Surgery with VTs is one of the most common ambulatory surgeries performed in children [4]. In Norway, approximately 6700 children undergo surgery annually [5].

Postoperative controls may continue for two or more years, and is needed to assess whether the tubes are functional, evaluate whether hearing loss has been improved, and to handle potential complications properly [6]. Complications after insertion of VTs include otorrhea, occlusion of tubes, premature extrusion, persistent perforation in the tympanic membrane, tympanosclerosis, retraction pocket, and cholesteatoma [6–8]. A meta-analysis concluded, however, that sequelae after VTs are generally transient (e.g., otorrhea) or cosmetic (e.g., focal atrophy, tympanosclerosis) [8]. VTs that are not spontaneously expelled within 2–3 years are recommended to be removed surgically to avoid persistent perforations [9, 10].

In most countries, postoperative controls are carried out by the specialist health care, mostly otolaryngologists [6, 9]. Despite the large annual number of surgeries with VTs, there exist no evidence-based consensus regarding frequency and the level of expertise sufficient for the postoperative controls of the VTs to ensure a safe and sufficient follow-up of the patients, with long-term successful effect of the surgery as the final outcome [10–12]. A Dutch study found that most postoperative controls did not lead to any clinical interventions and therefore questioned the need for regular follow-ups [13]. The Swedish Council on Health Technology Assessment completed a systematic literature review on the documentation of VT treatment but did not reach a conclusion regarding optimal postoperative care of healthy children with inserted VTs [11]. The Norwegian Society of Otorhinolaryngology, Head and Neck Surgery recommends that postoperative care is performed by otolaryngologists [9]. Consequently, otolaryngologists are doing most of the postoperative controls in Norway, but occasionally this is done by GPs [14].

In a previous retrospective study, we found no difference in hearing thresholds and middle-ear function 2 years after VT surgery, between children receiving postoperative care by otolaryngologists and by their regular GPs [15]. However, the low sample size and lack of randomization made it difficult to draw any final conclusions on whether postoperative care performed by GPs represent a safe alternative to otolaryngologists regarding the clinical outcome. Moreover, we did not examine complication rate, HRQoL of the children, or the parents’ evaluations of the postoperative care. To confirm or reject previous findings with a higher level of scientific evidence, we decided to carry out a larger randomized non-inferiority study.

The aim of our new study “Control of Ventilation Tubes” (ConVenTu) is to evaluate whether postoperative controls after VT surgery performed by GPs represent a safe and sufficient alternative to controls performed by otolaryngologists in otherwise healthy children. The optimal outcome of the treatment is that the child recovers with completely normal hearing, middle ear function, and healing of the tympanic membrane [6, 10]. We will therefore compare pure tone average (PTA) of hearing thresholds 2 years after surgery between children, who receive postoperative controls of VTs by otolaryngologists and by their GPs, respectively. We will also compare middle ear function, complication rate, HRQoL of the children, and the parents’ evaluations of the postoperative care. Results from this study may be utilized for deriving evidence-based clinical practice guidelines on the level of postoperative care after surgery with VTs. The aim of this protocol is to describe the ConVenTu study, which is the first randomized non-inferiority study of GPs performing postoperative controls after VT surgery.

## Methods

### Study design

**Fig. 1:**
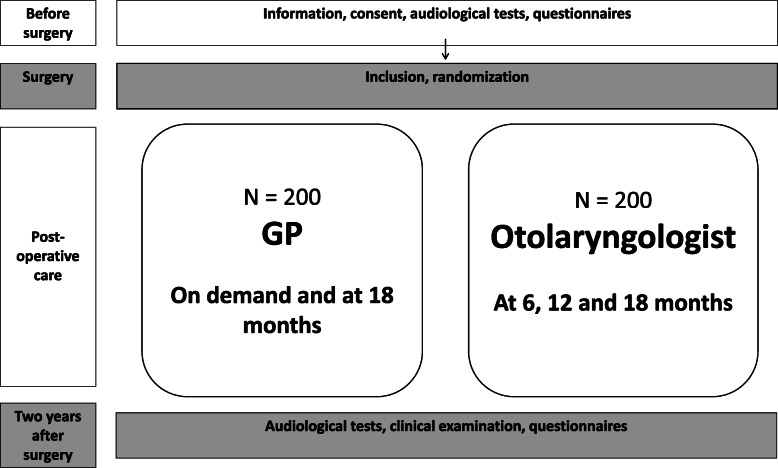
Flowchart

**Fig. 2:**
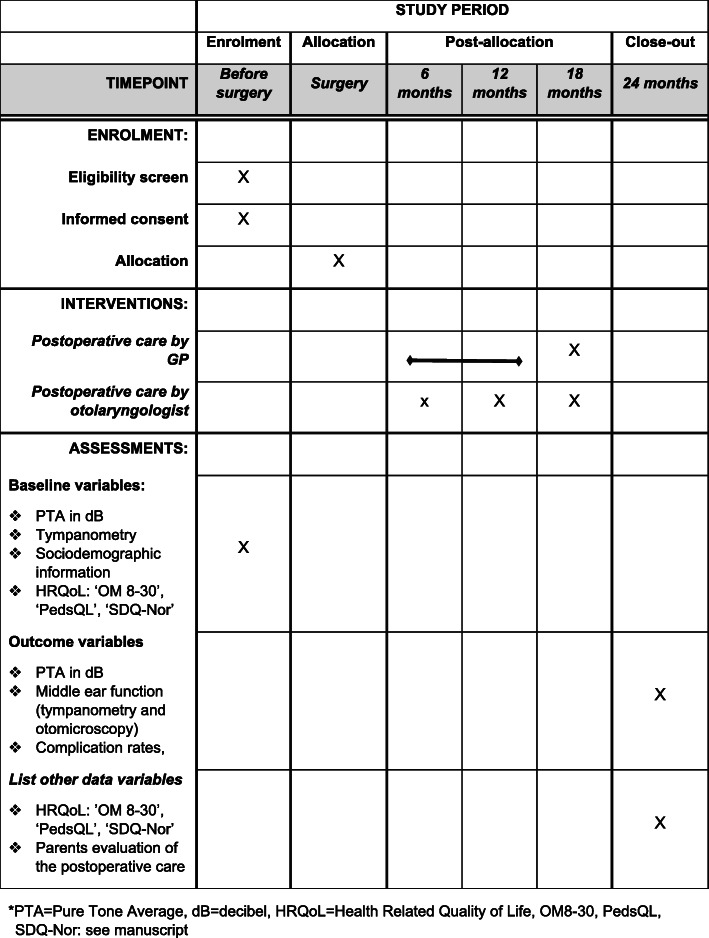
Schedule of enrollment, interventions, and assessments. PTA, pure tone average; dB, decibel; HRQoL, health-related quality of life; OM8-30; PedsQL, SDQ-Nor: see manuscript

The ConVenTu study is a multicenter randomized non-inferiority study conducted in clinical settings of seven hospitals located in all four Regional Health Authorities in Norway. The postoperative care is assessed by either an otolaryngologist at the hospital where VT surgery was performed, or by the patients’ regular GP. A flowchart of design and timeline in the study is presented in Fig. 1, and a SPIRIT schedule of enrollment, interventions, and assessments in Fig. 2, and trial registration data in Table 1.

**Table 1:** Trial registration data

Data category	Clinical data
Primary registry	ClinicalTrials.gov NCT02831985
Secondary identifying numbers	REC Mid-Norway 2015/902
Contact	bjarne.austad@ntnu.no
Short title	The ConVenTu study
Scientific title	Postoperative controls of ventilation tubes in children by general practitioner or otolaryngologist? Study protocol of a multicenter, randomized controlled trial (The ConVenTu study)
Country of recruitment	Norway
Population	Children 3–10 years after surgery with VTs
Study type	Multicenter randomized, non-inferiority study. Randomized into postoperative controls by either otolaryngologists or GP
Primary purpose	To evaluate whether postoperative care after surgery with VTs performed by GPs represent a sufficient alternative to otolaryngologists
First enrollment	August 2017
Target sample size	400
Primary endpoint	Hearing thresholds 2 years after surgery, measured as pure tone average
Secondary endpoint	Two years after surgery: Middle ear function assessed with tympanometry and otomicrocopy Complication rates Disease-specific and generic health-related quality of life Guardians evaluations of the postoperative care

### Eligibility criteria

Eligible participants are children, age 3–10 years, where VTs are placed in one or two ears. Exclusion criteria are conditions that need closer postoperative care by otolaryngologists: medical syndromes (e.g., Downs syndrome), other coexisting severe diseases, or severe neurogenic hearing loss in at least one ear (children with > 50 dB hearing threshold in at least one frequency 0.25–4.0 kHz are excluded). Also, children with cognitive impairment or no comprehension of Norwegian language are excluded.

### Random allocation of level of expertise for postoperative control of VTs

After discharge from the hospital, each patient who agreed to participate is randomly allocated to receive postoperative care by either an otolaryngologist or by their regular GP (Fig. 1). The randomization procedure is computerized and carried out by means of the “WebCRF” software developed at Unit for Applied Clinical Research at The Faculty of Medicine and Health Sciences, NTNU [16]. The design is block randomization with varying size of blocks, with an allocation ratio of 1:1, stratified on the study center.

### Procedures for postoperative controls

Children randomized to postoperative care by otolaryngologists will receive an appointment at 6, 12, and 18 months after surgery, to reflect the existing management of these patients in Norway. Postoperative care by GPs will not be planned appointments, but on the initiative of the parents as this reflects existing management in general practice. Parents of children randomized to the GP group will receive written information on when they need to contact their GP, for instance, if the child is suffering from otorrhea, persistently reduced hearing, or otitis media. The GPs receive information regarding the study and a procedure for postoperative care on how to treat the most common complications. In addition, a procedure for handling complications is enclosed in the hospital discharge letter after surgery; therefore, it is available for the GPs at point-of-care. If needed, the GP can refer the children to an otolaryngologist. If the VTs have not been spontaneously expelled within 18 months, the GP is requested to refer the patient to an otolaryngologist. Accordingly, the parents are told to contact the GP at the latest 18 months after surgery (Fig. 1).

### Audiological tests and assessment of sociodemographic factors

All participants will be evaluated with audiological tests prior to surgery and 2 years following surgery. Audiological tests are performed with audiometry and tympanometry at the hospital where the surgery takes place. Hearing thresholds are measured by pure tone audiometry. In the youngest children, equipment for play audiometry is used if needed [17]. Results from at least three of the pure tone thresholds in dB at 0.5, 1, 2, 4 kHz form the pure tone average (PTA), the main clinical outcome in this study. Two years after surgery, an otolaryngologist will examine all participants. Complications during the postoperative period will be registered by carefully examining the patient record.

Sociodemographic information is assessed through questionnaires at enrollment. Both before and 2 years after surgery, the parents are asked to complete questionnaires regarding their perception of the child’s HRQoL. The parents’ evaluation of the postoperative controls is assessed through questionnaires at the end of the follow-up period. Each study center has a local coordinator responsible for a Case Report Form to ensure complete recording of individual data.

### Primary and secondary endpoints

Hearing thresholds 2 years after surgery, measured as PTA, is the main clinical outcome and defined as the primary endpoint.

The following clinical and sociodemographic factors are considered as secondary endpoints:

1. Middle ear function assessed with tympanometry and otomicroscopy [18]
2. Complication rates
3. Disease-specific HRQoL by the otitis media questionnaire for children (“OM8-30”) [19]
4. Generic HRQoL by “PedsQL,” based on proxy-report (participants aged 3–8 years) or by adolescent self-report (8–12 years) and “SDQ-Nor” [20, 21]
5. Parents evaluation of the postoperative care

The secondary endpoint measures no. 1–2 act as surrogate markers for possible reduced hearing thresholds in the future. The secondary endpoint measures no. 3–5 are assessing the risks and benefits of postoperative care and important for patient-centered care.

### Project organization

The Department of Otolaryngology, Head and Neck Surgery at St. Olavs Hospital is the main investigator center and is responsible for coordination with the other participating hospitals. The study is organized in collaboration with the Norwegian University of Science and Technology (NTNU).

The steering committee is responsible for the implementation and progress of the study, applications, and data management. The principal investigator, the study director, and the main project coordinator are members of the steering committee and have regular communication. The reference group supports the decision-making and governance processes of the study. We have regular internal trial audits from the trial sponsor at the main investigator center. Study visits have been performed at all study sites to identify and overcome reasons which could hinder recruitment. In addition, meetings with all participating hospitals and user representatives are held bi-annually.

All handling of personal data will be in accordance with the EU General Data Protection Regulation (GDPR), as implemented at NTNU and St. Olavs hospital. All personal information will be anonymized. The anonymized data is only available for the steering committee. Processing of personal study audiological data will be done according to procedures approved by the data protection official at each study center. The nurses at the outpatient clinics are responsible for taking informed consent. The main project coordinator does the final check-up that all informed consents are taken and that they are stored in a secured place.

### Sample size and power

In this non-inferiority study, we defined equality in PTA 2 years after surgery (primary endpoint) as < 5 dB (5 dB as equivalence margin). Hearing thresholds are determined in 5 dB steps, and we also consider 5 dB as the minimum difference of clinical importance [22]. To avoid an incorrect conclusion of no difference between the groups (type II error), the power of the tests for detecting a difference of ≥ 5 dB must be high (≥ 95%, error margin < 5%). Thus, with a power of 95% and a significance level of 5% (two-sided test), 105 participants are needed in each group to be able to detect an absolute difference in mean PTA between the groups of ≥ 5 dB (standard deviation of 10 dB in each group). With an additional 15% for dealing with a potential skew distribution of hearing level, and a 20% further increase to account for potential dropouts during the follow-up period, we are left with a sample size of 145 participants in each group. To maintain power in an analysis stratified for seven study centers (six additional parameters in model), the total sample size must be increased by at least 60 participants, applying general rules of thumb of at least 10 cases per parameter. If we assume that the 60 participants are evenly distributed in the two randomization groups, this leaves us with a sample size of at least 175 participants in each group. We chose to truncate upwards to 200 participants in each group (Fig. 1) to maintain power in case of a higher dropout rate or a need for inclusion of additional adjustment factors.

### Statistical analysis

The analyses will be performed according to intention-to-treat. Unadjusted two-sample *T* tests, and a general linear model and/or a linear mixed model (LMM), with study center as fixed or random factor, respectively, will be applied to compare mean PTA 2 years after surgery, to quantify the effect of the treatment and to validate results in case of potential inequalities between the two randomization groups. Furthermore, we will also compare the mean change in PTA after 2 years (change from baseline). Similar analytic methods will be applied for other relevant variables that are measured on a continuous scale (HRQoL). Log-transformation, or non-parametric methods, may need to be considered. Generalized linear mixed model and/or McNemar’s test is relevant for analyzing changes in categorical, dichotomous outcome variables. A chi-square test, or Fischer exact test, is relevant for comparing the two study groups with respect to the number of complications during postoperative care. Equality between the two treatment groups will be evaluated in terms of the magnitude of observed differences (point and interval estimate).

## Discussion

Insertion of VTs is among the most common surgical procedures in childhood and postoperative care can take several years and is usually reviewed by otolaryngologists [4]. Evidence concerning which level in the health care system is sufficient to handle the postoperative controls is lacking [9–11, 13]. Possible disadvantages of postoperative care carried out by GPs could be a lack of knowledge and equipment and failure to handle possible complications. On the other hand, possible benefits could be shorter travel distance, increased flexibility of time point of controls, better continuity of care, and possibly lower costs [23]. It is therefore important to evaluate whether postoperative care performed by GPs represents a safe and sufficient alternative to otolaryngologists concerning the clinical outcome of the treatment.

One of the major strengths of the ConVenTu study is that it is designed to detect a small difference in hearing thresholds (≥ 5 dB). As far as we know, there is no consensus regarding what should be considered as the minimum clinical important difference in hearing threshold. Chow et al. defined 10 dB as being of clinical importance [24]. Because our study affects children, our equality margin is conservative, only 5 dB. In clinical practice, hearing thresholds performed with audiometry is measured in 5 dB steps, and this is thus the lowest difference that can be detected. To avoid to incorrectly conclude that postoperative control performed by GPs is a sufficient level of expertise for postoperative controls of VTs, the statistical power of the test for detecting a difference in hearing threshold larger than our equality margin was very high (95%). Another strength of our study is that it also includes evaluation of middle ear function and complication rates, HRQoL of the children, and the parents’ evaluation of the postoperative care, during a follow-up prolonging for 2 years. Moreover, participants are recruited from all different regions of Norway, to cover potential variation in clinical practice.

A limitation of our study is that it covers only ages 3–10 years, although many other younger children receive VTs [25]. The reason for not including younger children is the difficulties to achieve reliable audiological tests in children < 3 years [26]. Moreover, we do not assess complications after surgery with VTs that occur later than 2 years. However, a 14-year prospective follow-up study after the insertion of VTs concluded that tube insertion is mostly a safe and useful treatment method [27].

The ethical concern designing this study relates to a potentially worse outcome after surgery in children randomized to postoperative care by GPs. Therefore, all participants are thoroughly examined with audiological tests and by an otolaryngologist 2 years after surgery. There is no summoning to the recommended postoperative controls in general practice, and there is a risk that parents do not book an appointment when needed despite the information given. However, in our previous retrospective study with a similar design, a lack of attendance in the GP group was not a problem [15]. Moreover, this patient group is in general healthy except for their ear problems. Children with an expected increased risk of complications, with severe neurogenic hearing loss or other severe coexisting diseases, are not considered for inclusion. If the GP finds that the child needs follow-up by a specialist, he or she will refer the child to an otolaryngologist. The Ethical Committee that reviewed the study did not have any comments on this procedure.

The setting for this study is Norwegian healthcare system where nearly every citizen has one specific GP [28]. Nevertheless, we believe our findings will be of general interest, since surgery with VTs is one of the most common surgical procedure among children.

### Trial status

This trial started the inclusion of study participants in August 2017 and the recruitment period was then estimated to be 3 years. However, the recruitment of study participants has been slower than expected, partly due to a lower number of children above 3 years in need of VT, and partly due to the COVID19 pandemic (from March 2020). Thus, the data recruitment period must be extended to reach our target of 400 participants. The follow-up period is scheduled to 2 years, and the data collection phase is expected to be completed in fall 2023. All personnel who have contributed significantly with the planning and performance of the study may be included in the list of authors in accordance with the Vancouver rules [29] The results of this study will be submitted for publications in peer-reviewed journals and communicated to the participants and the Ethics Committee according to EU and national regulations. It is our intention with this study to come up with new knowledge regarding optimal postoperative care after surgery with VTs.

## Data Availability

The datasets generated during and/or analyzed during the current study are not publicly available due Norwegian regulations concerning sensitive data but are available from the corresponding author on reasonable request.

## Abbreviations

VT: Ventilation tube
GP: General Practitioner
HRQoL: Health-related quality of life
PTA: Pure tone average
ConVenTu: Control of Ventilation Tubes
