# Correction: Postoperative controls of ventilation tubes in children by general practitioner or otolaryngologist? Study protocol for a multicenter randomized non-inferiority study (The ConVenTu study)

**DOI:** 10.1186/s13063-024-08259-7

**Published:** 2024-06-20

**Authors:** Bjarne Austad, Ann Helen Nilsen, Anne-Sofie Helvik, Grethe Albrektsen, Ståle Nordgård, Wenche Moe Thorstensen

**Affiliations:** 1 General Practice Research Unit, Department of Public Health and Nursing, Norwegian University of Science and Technology (NTNU), Trondheim, Norway; 2 Øya Medical Centre, Trondheim, Norway; 3 Department of Otolaryngology, Head and Neck Surgery, St. Olavs Hospital, Trondheim, Norway; 4 Department of Neuromedicine and Movement Science, NTNU, Trondheim, Norway

**Correction** **:** **Trials 21, 950 (2020)**

**https://doi.org/10.1186/s13063-020-04849-3**

Following the publication of the original article [1], we have been notified that that the below sentence marked in bold needs to be removed from the Eligibility criteria of the Methods section:

“Exclusion criteria are conditions that need closer postoperative care by otolaryngologists: medical syndromes (e.g., Downs syndrome), other coexisting severe diseases, or severe neurogenic hearing loss in at least one ear (children with > 50 dB hearing **threshold in at least one frequency 0.25–4.0 kHz are excluded**).”
